# Crosstalks between Cytokines and Sonic Hedgehog in *Helicobacter pylori* Infection: A Mathematical Model

**DOI:** 10.1371/journal.pone.0111338

**Published:** 2014-11-03

**Authors:** Shruti Marwaha, Michael A. Schumacher, Yana Zavros, Hamid R. Eghbalnia

**Affiliations:** Department of Molecular and Cellular Physiology, University of Cincinnati, Cincinnati, Ohio, United States of America; Indian Institute of Science, India

## Abstract

*Helicobacter pylori* infection of gastric tissue results in an immune response dominated by Th1 cytokines and has also been linked with dysregulation of Sonic Hedgehog (SHH) signaling pathway in gastric tissue. However, since interactions between the cytokines and SHH during *H. pylori* infection are not well understood, any mechanistic understanding achieved through interpretation of the statistical analysis of experimental results in the context of currently known circuit must be carefully scrutinized. Here, we use mathematical modeling aided by restraints of experimental data to evaluate the consistency between experimental results and temporal behavior of *H. pylori* activated cytokine circuit model. Statistical analysis of qPCR data from uninfected and *H. pylori* infected wild-type and parietal cell-specific SHH knockout (PC-SHH^KO^) mice for day 7 and 180 indicate significant changes that suggest role of SHH in cytokine regulation. The experimentally observed changes are further investigated using a mathematical model that examines dynamic crosstalks among pro-inflammatory (IL1β, IL-12, IFNγ, MIP-2) cytokines, anti-inflammatory (IL-10) cytokines and SHH during *H. pylori* infection. Response analysis of the resulting model demonstrates that circuitry, as currently known, is inadequate for explaining of the experimental observations; suggesting the need for additional specific regulatory interactions. A key advantage of a computational model is the ability to propose putative circuit models for *in-silico* experimentation. We use this approach to propose a parsimonious model that incorporates crosstalks between NFĸB, SHH, IL-1β and IL-10, resulting in a feedback loop capable of exhibiting cyclic behavior. Separately, we show that analysis of an independent time-series GEO microarray data for IL-1β, IFNγ and IL-10 in mock and *H. pylori* infected mice further supports the proposed hypothesis that these cytokines may follow a cyclic trend. Predictions from the *in-silico* model provide useful insights for generating new hypothesis and design of subsequent experimental studies.

## Introduction

*Helicobacter pylori* is a gram negative bacteria that resides in the stomach and duodenum of infected host [1], [2]. It is a significant risk factor for atrophic gastritis [3], [4], gastric ulcer [4], [5] and gastric cancer [4]–[6]. Improved treatment and early diagnosis of *H. pylori* associated diseases require a better understanding of the mechanisms by which this bacteria increases the risk for chronic inflammation and gastric cancer [7]. The host cell detects the presence of bacteria and produces an immune response to eliminate the bacteria. The final outcome depends on a balance between pro-inflammatory and anti-inflammatory cytokines produced during *H. pylori* infection [8], [9]. A strong pro-inflammatory response may allow eradication of the bacteria however at a cost of increased risk for gastritis [10] while anti-inflammatory cytokines may protect against gastritis, but *H. pylori* may continue to persist [11], [12]. Another gene that has been recently identified to play an important role in pathogenesis of chronic *H. pylori* infection [13], [14] and gastric cancer [17]–[20] is sonic hedgehog (SHH). In both human and mouse stomachs, it is expressed in the parietal cells [21]. Under normal conditions, SHH regulates differentiation of the gastric epithelium [21], [22] and T-cell [23], [24]. During chronic *H. pylori* infection, SHH-dependent proliferation of parietal cells plays a key role in gastric mucosal repair [13], [25]. It is also reported that SHH acts as a monocyte/macrophage chemoattractant during repair of the myocardium [26] and during *H. pylori* infection [15]. We and others have recently shown that SHH is involved in early immune response to *H. pylori* [15], [16]. We observed that parietal cell-specific SHH knock-out (PC-SHH^KO^) mice failed to develop gastritis, even after 6 months of *H. pylori* infection in contrast to infected control group (WT) which developed significant inflammatory response [15]. Zaatari et al have shown that overexpression of Shh^WT^ induced gastritis while CMV-Shh^F200H^ mice (carrying mutant SHH) did not develop gastritis. They also reported that SHH overexpression exacerbated the histologic severity observed with *Helicobacter felis* infection and increased the amount of myeloid cells recruited to the inflamed stomach as compared to that in non-transgenic mice [16].

Although recent studies have highlighted the immunoregulatory role of SHH in stomach [15], [16], a model studying temporal relationship between SHH and cytokines activated during *H. pylori* infection is still lacking, and it is unexplored what effect SHH may have in the context of regulating cytokine expression in the *H. pylori* infected stomach. Such temporal studies are not readily amenable to experimental approaches because of high cost and time associated with time series in-vivo experiments. In-vitro experiments of immune responses often face experimental limitations – for example, lack of a host immune cell response. Mathematical modeling is a powerful technique to complement such studies as it allows predicting dynamic behavior of the system under various perturbations and generating new hypotheses. Currently, there are only a handful of mathematical models that study the *H. pylori* - host immune response, and though they provide high-level or cellular-level details [27]–[30], they do not focus on quantifiable biomarkers involved.

An interaction map - a topological network of signaling pathways, activated by *H. pylori*, was manually curated from literature [31], [32] (Figure S1 in Methods S1). *H. pylori* virulence factors like CagA [33]–[35], VacA [33] and PGN [33] activate a cascade of signaling pathways in epithelial cells of host stomach. Virulence factor CagA (cytotoxin-associated gene A) activates ERK [25], [34], [36] and AKT [37] pathways which further stimulate nuclear translocation of NFĸB [37]–[39]. Effector molecule PGN (peptidogylcan) is sensed by intracellular receptor NOD1 (nucleotide-binding oligomerization domain 1) [25], [33] which activates NFĸB [25], [33], [35], [40], ERK, p38 and AP-1 [40]. Protein VacA (Vacuolating cytotoxin A) also stimulates ERK and p38 pathways [41] which activate transcription factor AP-1 [34], [42]–[44]. Both NFĸB and AP-1 positively regulate chemokine IL-8/MIP-2 transcription [34], [35], [45]. (MIP-2 is a functional homolog of human IL-8 in mouse [46]). *H. pylori* colonization of gastric epithelium eventually leads to recruitment of monocytes which secrete pro-inflammatory cytokines like IL-12, IL-1β, TNFα, IL6 [47]–[49] and IL8 [50], [51]. IL-12 and other *H. pylori* antigens synergistically stimulates release of IFNγ from natural killer cells [49], [52]–[54]. SHH, a crucial player in early immune response to *H. pylori* [15] has been reported as a putative target of NFĸB in gastric [55] and pancreatic cancer [56], [57]. Interestingly, Waghray *et al* have shown that IL-1β suppresses SHH expression in parietal cells by inhibiting acid secretion [58]. Along with the activation of pro-inflammatory cytokines, IL-10, an anti-inflammatory cytokine is also produced during *H. pylori* infection [47], [54], [59]. IL-10 has been reported to inhibit NFĸB activity, and subsequently IL-8 transcription in gastric epithelial cells during *H. pylori* infection [60] and also in macrophage [61] and T-cells [62]. In summary, different virulence factors of *H. pylori* activate various signaling pathways in the stomach tissue leading to increased activity or expression of NFĸB, chemokines and cytokines [63], [64]. The severity of inflammation and exact details of signaling pathways described above can vary depending on the strain of *H. pylori* and strain of mice used in the experiment [65], [66].

The role of SHH in *H. pylori* mediated inflammation was investigated experimentally using wild-type and parietal cell-specific SHH knock-out (PC-SHH^KO^) mice [15]. Here we investigate, using the experimental data and mathematical modelling technique, the putative dynamics of cytokine-SHH circuitry. Our analysis demonstrates an interaction between *H. pylori* infection and genotype of the mice, and positive regulation of the cytokines' expression involving SHH. A detailed examination of the current pathway databases (for example Strings, Pathway Commons), and molecular circuits (wiring diagram of interactions among genes and proteins) derived through manual curation of current literature, fail to identify the necessary positive regulation. Our mathematical model of the cytokine-SHH circuitry aims to understand temporal behavior of the network and to identify emergent patterns which may arise through interactions among components of the system and may not be apparent when the components are studied in isolation [67]–[69].

## Materials and Methods

### Animal Model

The mouse model with parietal cell-specific deletion of SHH (PC-SHH^KO^) was generated as previously described (C57Bl/6, 129/Sv background) [22] and HKCre mice expressing Cre transgene under the control of H^+^,K^+^-adenosine triphosphatase (ATPase) β subunit promoter (C57Bl/6, FVB/N background) were used as control [15]. HKCre (WT) and PC-SHH^KO^ mice, aged 8 weeks were either infected with *H. pylori* or left uninfected. The uninfected group received 200 µl of Brucella broth over 3 consecutive days whereas the infected group was inoculated with 10^8^ *H. pylori* SS1 (Sydney strain 1) bacteria per 200 µl of Brucella broth over 3 consecutive days. Mice (n = 3–4 per group) were sacrificed on day 7 and 180 post-infection and levels of gastric SHH and cytokines were assayed by quantitative reverse-transcriptase polymerase chain reaction. *H. pylori* colonization as measured by bacterial cultures and analysis of CFU/g Tissue (colony-forming units per gram tissue) for WT and PC-SHH^KO^ infected mouse stomachs were shown to be equivalent [15].

### Ethics Statement

All mouse studies were approved by the University of Cincinnati Institutional Animal Care and Use Committee (IACUC) that maintains an American Association of Assessment and Accreditation of Laboratory Animal Care (AAALAC) facility.

### Quantitative real-time RT-PCR (qPCR)

Total RNA was isolated from stomachs of uninfected and infected WT and PC-SHH^KO^ mice. The High Capacity cDNA Reverse Transcription Kit was used for cDNA synthesis from 100 ng of RNA following the recommended protocol (Applied Biosystems). Pre-designed real-time PCR assays were purchased for the following genes (Applied Biosystems): SHH (Mm00436528_m1), IFNγ (Mm01168134_m1), IL-1β (Mm01336189_m1) and mouse GAPDH (20X) (4352932-0803020), MIP2 (Mm00436450_m1). PCR amplifications were performed in a total volume of 20 µl, containing 20X TaqMan Expression Assay primers, 2X TaqMan Universal Master Mix (Applied Biosystems, TaqMan Gene Expression Systems) and cDNA template. Each PCR amplification was performed in duplicate wells in a StepOne Real-Time PCR System (Applied Biosystems), using the following conditions: 50°C 2 minutes, 95°C 10 minutes, 95°C 15 seconds (denature) and 60°C 1 minute (anneal/extend) for 40 cycles. IL-12 expression was quantified using specific primers as previously published for IL-12: Forward- 5′- GGA AGC ACG GCA GAA TA-3′ and Reverse- 5′- AAC TTG AGG GAG AAG TAG GAA TGG -3′ [70] using the SYBR Green PCR Master Mix and protocol (Applied Biosystems). GAPDH was used as an internal control. See Methods S1.

### Statistical Analysis

For each sample, dCT value for target gene was calculated by subtracting CT value of calibrator gene (GAPDH) from CT value of target gene. To test if expression of target gene in gastric mucosa was affected by *H. pylori* and if this effect was same in all genotypes, a two-way ANOVA (Analysis of variance) test was performed, comparing dCT values from all four condition: uninfected wild type, infected wild type, uninfected PC-SHH^KO^ and infected PC-SHH^KO^. Bonferroni test was performed as post ANOVA test to assess the effect of *H. pylori* on target genes in each genotype. dCT values from uninfected mice were compared with dCT values from *H. pylori* infected mice in both WT and PC-SHH^KO^ conditions. For graphical presentation of qPCR results, for each gene in WT and PC-SHH^KO^ conditions, ddCT was calculated by subtracting dCT value of *H. pylori* infected group from dCT of uninfected group. The data were plotted as 2^−ddCT^ (mean fold change) [12]. A P-value <0.05 was considered statistically significant. Interaction test was also performed to study the interaction between infection status and genotype. Since CT value is inversely proportional to gene's expression level, negative dCT values were used for interaction test. The interaction plot displays levels of treatment (absence and presence of *H. pylori*) on the x-axis and mean of negative dCT values for each treatment on the y-axis. A separate line connects the means corresponding to each level of the trace factor – genotype (WT and PC-SHH-KO). The qPCR data was analyzed using R statistical software. Link to the R script used for analyzing the data and generating graphs: http://rpubs.com/marwahsi/20168.

### Mathematical model

**Figure 1:**
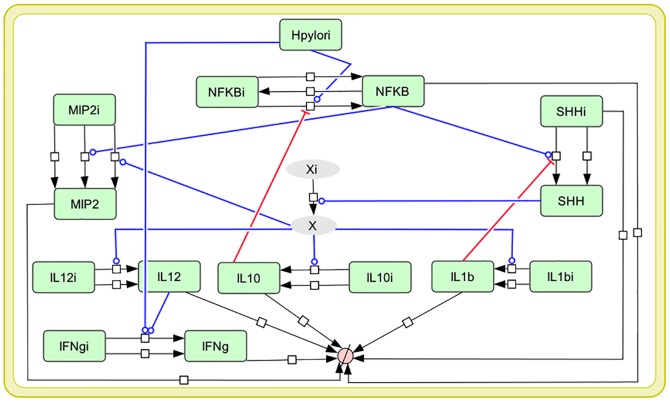
Diagram of mathematical model of cytokine-SHH network during *H. pylori* infection. This reduced network derived from interaction map, represents the key cytokines activated as host's immune response to *H. pylori*. Blue arrows represent activation whereas while red arrows depict inhibition. Model species with suffix “i” represent the inactive form. The link between SHH and cytokines, as predicted by our experimental data is modelled through unknown model species “X” (grey colored). Detailed interaction network of host immune response to *H. pylori* is available in MethodsS1.

A comprehensive interaction map was developed by manual curation of published literature, capturing key signaling pathways activated in gastric epithelium and in macrophages by *H. pylori* virulence factors. For our mathematical model, a selected subset of biomolecules from the interaction map was included. The molecules were selected for inclusion if: a) they were reported in the literature to be regulated with *H. pylori* SS1 strain (as SS1 strain was used in our experiments) [63], [64] or b) their experimentally measured levels showed variations in our data. Mathematical modelling of very large networks can be impractical, primarily due to unavailability of parameter values, especially in case of higher organisms [31], [71]–[73]. In order to overcome these challenges, the complex interaction map was pruned to a simpler reduced network (Figure 1) focusing on cytokines and SHH, for which experimental trends were available [74]. The reduced model can capture key characteristics of the larger network and conserve the regulatory mechanisms present in the system [71]–[73]. Our reduced model compresses the details of a complex pathway into a “black box”, which can be scaled up, by adding detailed components as needed. The reduced model was further enriched with a new “influence” link between SHH and cytokines as predicted by our experimental data. It must be noted that this link may not be a direct interaction between SHH and cytokines and rather may involve an indirect mechanism. This unknown mechanism is currently shown by an intermediate species “X” in the model which links SHH to all the cytokines. Equations based on Michaelis-Menten and mass action kinetics were used in the reduced model in order to construct a dynamic system that describes the evolution of the biomolecules over time. The sampling frequency (control equaling time zero, day 7 equaling early, and day 180 equaling late) was unlikely to be sufficient for obtaining detailed dynamics of concentration and oscillation frequencies. Therefore, unit-free measures were used to express “time” and “amount” in order to focus on the *qualitative behavior* of the cytokine dynamics. The kinetic parameters initially selected for the model, represent a range of biologically feasible values [75]–[77]. Subsequent computational optimization (iterative trial and error) was used to select the parameter set that best satisfies the trends observed in experimental data and are within biologically relevant limits. This approach is similar to the methodology used for parameter estimation for computational models when no experimental values are available [76], [78], [79]. In addition to the reactions activated through *H. pylori* signaling, each model species is activated by a small “constitutive” flux which accounts for some basal levels of the species formed through a pathway either not represented in the model or currently unknown. Each model species is connected to a source and sink. The source represents inactive form of the protein. The sink accounts for downstream signaling and half-life of the protein. Complete list of model assumptions are available in Table S1 in Methods S1. Ordinary Differential Equations (ODE) were simulated to characterize the system using modeling tool CellDesigner [80]. Knock-out (KO) of different genes was modeled by setting the concentration of its source to zero. See Methods S1 for model equations and parameter values (Table S2, S3 and S4 in Methods S1).

### GEO Data

A time-series microarray dataset – GSE37938, available through GEO (Gene Expression Omnibus) database was used as an auxiliary data set in order to analyze the temporal behavior of the genes present in our model. The microarray data set contains six-week-old female BALB/c mice. The mice were uninfected or infected with SS1 strain of *H. pylori* for 2,7,14 & 28 days. A total of 71 samples (35 infected +36 uninfected) were collected from 3 cell types: chief cell, parietal cell and mucous producing pit cell [81]. We extracted and analyzed time-series data for IL-10, IFNγ, IL-1β from GSE37938 dataset. Data was not available for SHH and MIP-2 while IL-12 data was not used for analysis due to large number of missing values. For a given probe, median of its expression values was calculated for each time point. Probes with two or more missing values for more than one time point, were not considered for further analysis. The median values of a probe from both uninfected and *H. pylori* infected mice were plotted against time to study their trajectory in the two conditions. To address the problem of probe selection in case of multiple probes representing the same gene, median value was taken for probes with same Genbank ID (GB_list ID) while probes with different Genbank ID were analyzed separately. Plots are shown only for Genbank IDs which are represented by at least three probes. Link to the R script used for extracting and analyzing the data: http://rpubs.com/marwahsi/8932.

## Results

### SHH positively regulates cytokine expression during *H. pylori* infection

**Figure 2:**
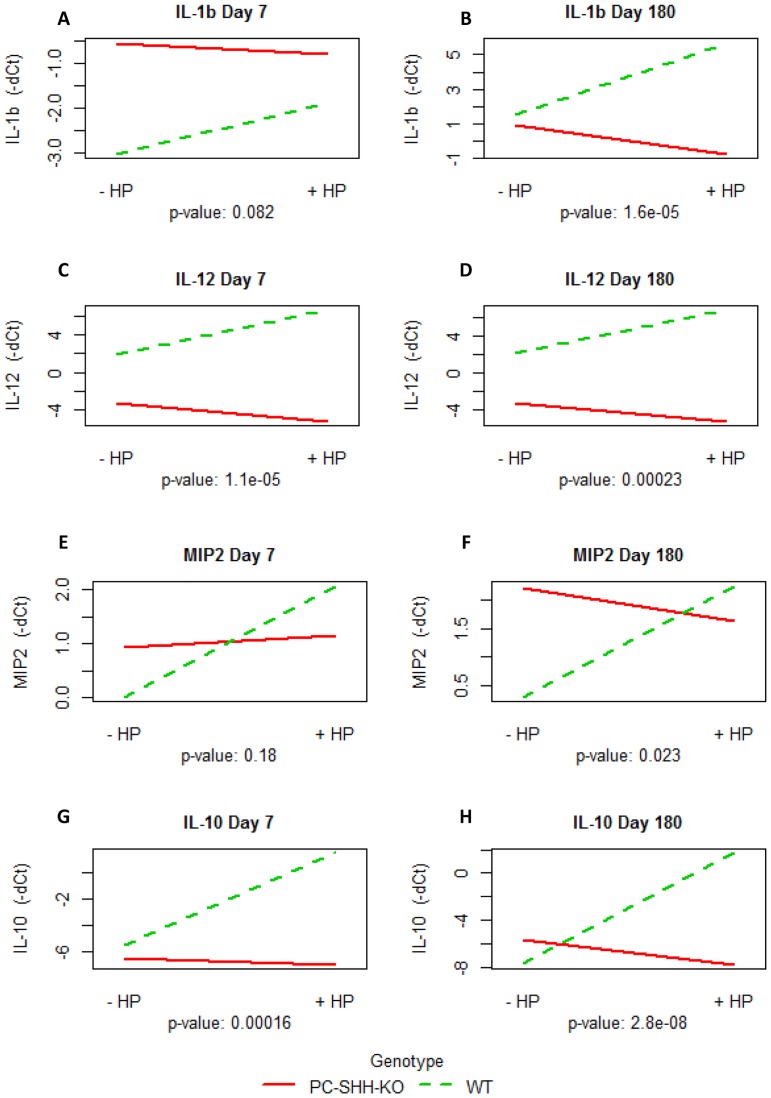
Interaction between infection status and genotype. RNA was extracted from stomachs of uninfected (-HP) and *H. pylori*-infected (+HP) wild type (WT) and parietal cell specific SHH knock out (PC-SHH-KO) mice 7 and 180 days post-inoculation. Expression of genes was measured by qPCR and interaction test was performed. Parallel lines imply that *H. pylori* has same effect on gene's expression in WT and PC-SHH^KO^ mice whereas intersecting or non-parallel lines indicate an interaction between genotype and infection. The graphs show interaction plot between infection status and genotype for (A) IL1β on day 7, (B) IL1β on day 180, (C) IL-12 on day 7, (D) IL-12 on day 180, (E) MIP-2 on day 7 (F) MIP-2 on day 180 (G) IL-10 on day 7 and (H) IL-10 on day 180. P-value for interaction between infection and genotype were calculated by two-way ANOVA test. Y-axis: mean of negative dCT value of cytokine, X-axis: infection status, trace-factor: genotype.

To determine if gastric SHH regulates expression of cytokines activated during *H. pylori* infection, WT and PC-SHH^KO^ mice were infected with Brucella broth or *H. pylori*, and cytokine expression was measured by qPCR in tissues collected from mouse stomachs, 7 and 180 days post inoculation. Interaction plots shown in Figure 2 (and Figure S2 in Methods S1) examines if *H. pylori* affects cytokine expression and if this effect is different in WT and PC-SHH^KO^ mice. Non-parallel lines that do not cross or crossing lines, imply an interaction effect between the two factors (genotype and infection status) [82]. An interaction effect between genotype and infection was observed for expression of all cytokines (Figure 2). Parallel lines would have implied that the effect of *H. pylori* on cytokine expression is the same in WT and PC-SHH^KO^ mice. On day 7, the interaction effect was statistically significant for IL-12 and IL-10 but not for IL-1β and MIP-2. For day 180, statistically significant interaction effect between genotype and infection was observed for all cytokines.

**Figure 3:**
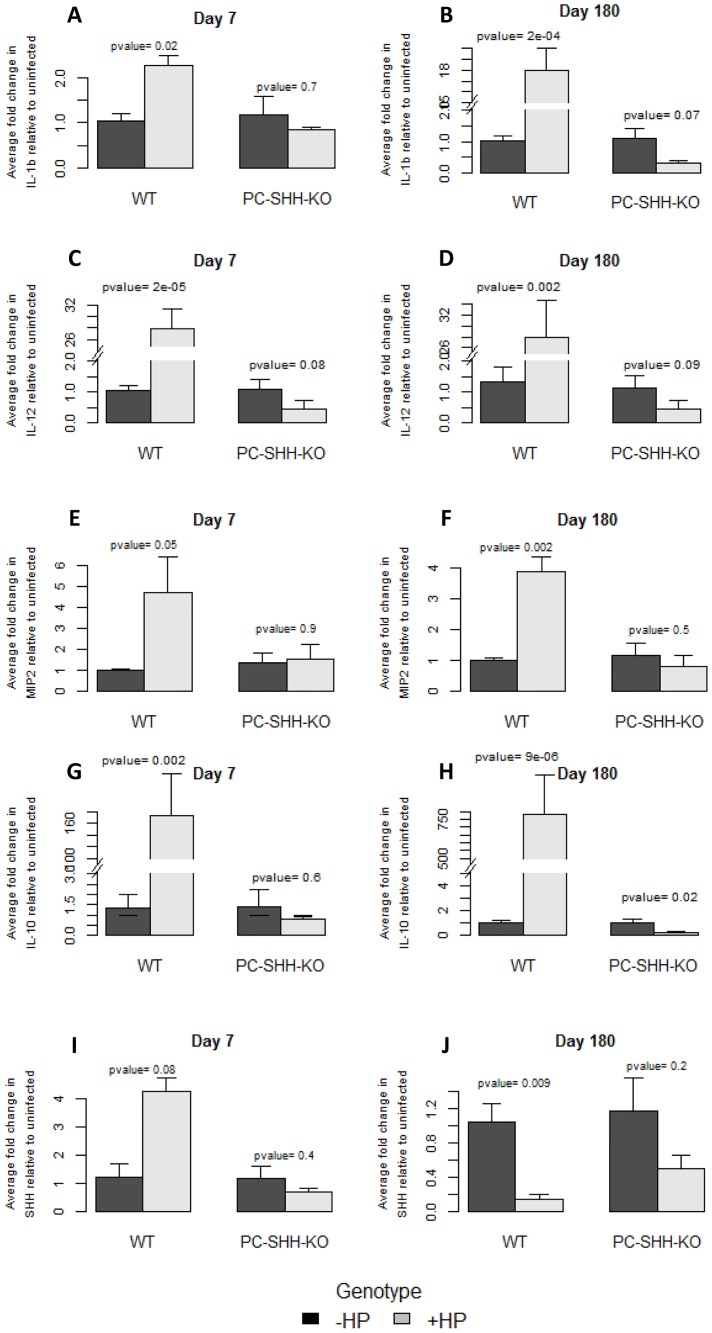
Effect of *H. pylori* on SHH and cytokines' expression in WT and PC-SHH^KO^ mouse stomachs, day 7 and day 180 post-inoculation. RNA was extracted from stomachs of uninfected and *H. pylori*-infected wild type (WT) and parietal cell-specific SHH knock out (PC-SHH-KO) mice 7 and 180 days post-inoculation. Expression of genes was measured by qPCR and two-way ANOVA test was performed, followed by Bonferroni test to compare uninfected (-HP) with *H. pylori* infected group (+HP) in each genotype. The graphs show average fold change in expression of IL-1β (A, B), IL-12 (C, D), MIP-2 (E, F), IL-10 (G, H) and SHH (I, J) upon *H. pylori* infection relative to uninfected condition. Bars represent the mean ± SEM, n = 3-4 per group.

Two-way ANOVA test was performed, comparing dCT values from all four conditions (WT+ Brucella Broth, WT + *H. pylori*, PC-SHH^KO^ + Brucella Broth, PC-SHH^KO^ + *H. pylori*), followed by Bonferroni test to compare specific groups (Figure 3 and Figure S2 in Methods S1). All cytokines showed a statistically significant increase with *H. pylori* in WT mice, on both day 7 and day 180 (except MIP-2 on day 7, which increased with a P-value of 0.05). However, this increase in cytokines' expression with *H. pylori* was not observed in PC-SHH^KO^ mice. These observations suggest the potential role of SHH in positive regulation of expression of IL-12, IL-1β, IL-10, IFNγ and MIP-2 during *H. pylori* infection. An unexpected trend was observed for IL-10 on day 180 in PC-SHH^KO^ mice - IL-10 expression was significantly lower in *H. pylori* infected mice as compared with uninfected mice on day 180, although it did not change significantly with *H. pylori* on day 7.

### Mathematical Model Behavior

An important goal of model building is to ensure that the model can recapitulate the experimental trends. SHH knock-out condition was tested in the model that is curated using current pathway databases and literature. This model lacks the putative positive regulation link (kcat9  = 0) suggested by our qPCR analysis. The model exhibited no change in levels of IL-12, IL-10, IL-1β, IFNγ or MIP-2 upon simulating SHH KO condition (Figure S3 in Methods S1). The visual results of simulation were mathematically verified by examining the eigenvalues of the Jacobian matrix. All eigenvalues were real and negative, which indicated a constant-level of all constituents at equilibrium. The experimental observation of diminished concentration of IL-10 on day 180 highlights the need to account for a missing regulatory link between SHH and cytokines.

**Figure 4:**
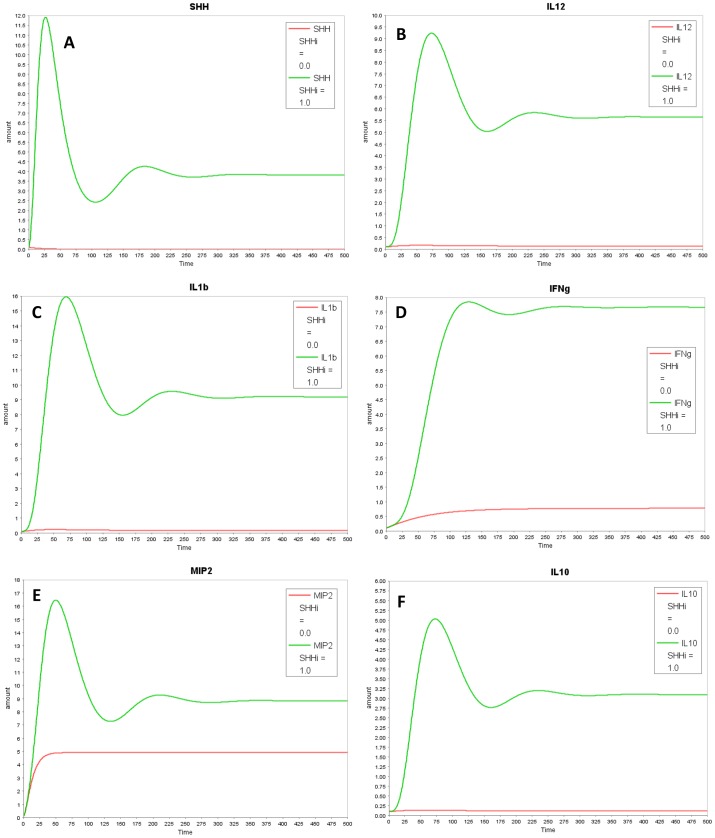
In-silico SHH KO results show a decrease in cytokines as comared to WT. SHH KO condition was simulated by setting SHHi to zero. Graph A–F shows profiles of (A) SHH (B) IL-1β (C) IL12 (D) IFNγ (E) MIP2 (F) IL10. Wild type condition (SHHi  = 1) is shown in green and *in-silico* SHH KO condition (SHHi  = 0) is represented in red.

**Figure 5:**
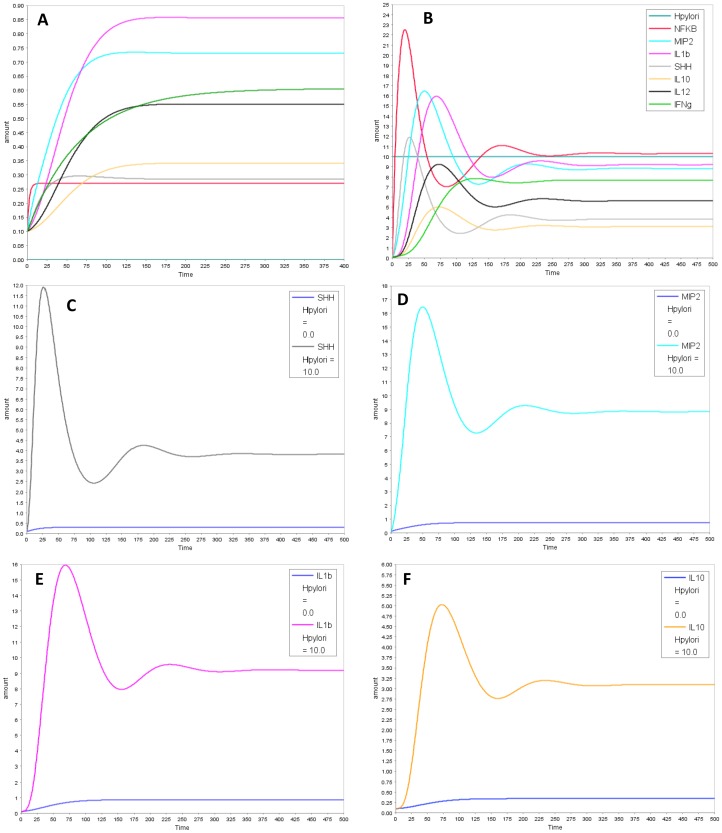
Temporal profiles of model species in uninfected and infected conditions. Simulation results comparing temporal profiles of model species in (A) absence and (B) presence of *H. pylori*. Graph C–F show temporal profiles of (C) SHH (D) MIP-2 (E) IL-1β and (F) IL-10 in absence and presence of *H. pylori*.

**Figure 6:**
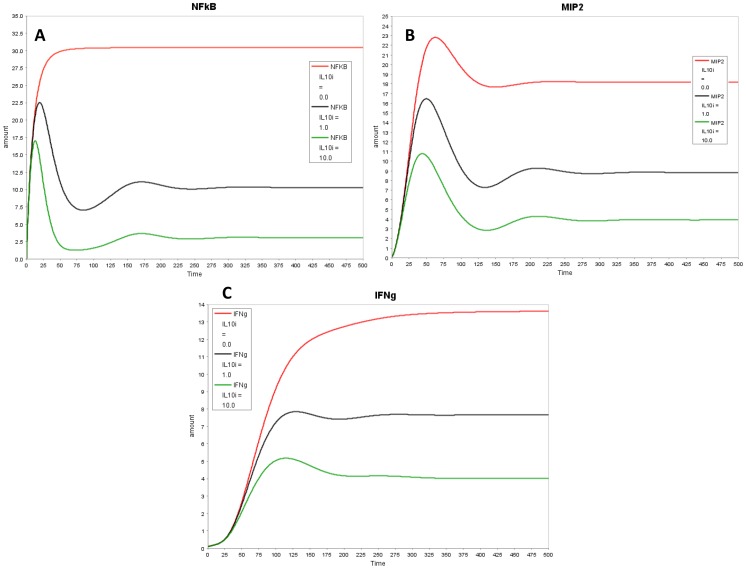
In-silico IL-10 knock-out and overexpression. Effect of IL-10 knock-out and overexpression on (A) NFkB (B) MIP-2 and (C) IFNγ. Wild type condition (IL10i  = 1) is shown in black, *in-silico* IL-10 knok-out (IL10i  = 0) in red and *in-silico* IL-10 overexpression (IL10i  = 10) in green.

In our model with the putative link, all cytokines: IL-12, IL-1β, IFNγ, IL-10 and MIP-2 decreased with SHH knock-out (Figure 4), aligning the model closer to the experimental results. The decrease in MIP-2 was not considerable as MIP-2 is also formed through a parallel pathway via NFĸB. Next, we assessed the effect of *H. pylori* on the temporal profile for all model species *in-silico* (Figure 5). The simulation results align qualitatively with the experimental trends of cytokines. All cytokines increased with switching *H. pylori* “on” in the model. Temporal profile for each model species is compared in the presence and absence of *H. pylori* in Figure 5 (MIP-2, SHH, IL-1β and IL-10) and Figure S4 in Methods S1 (IL-12, IFNγ). Dynamic modeling of the reduced network shows that with *H. pylori* stimulation, there is increase in NFĸB which transcribes MIP-2 and SHH. SHH activates IL-1β, IL-12 and IL-10 through unknown mechanism (shown through “X” in the model). IL-10 in turn inhibits NFĸB, forming a negative feedback loop. This inhibition decreases NFĸB which lowers SHH and eventually decreases IL-10 and other cytokines. The decrease in IL-10 relieves the inhibition on NFĸB, allowing it to rise again. These interactions between NFĸB, SHH and IL-10 form a negative feedback loop and give rise to damped oscillations in the model (Figure 5B). Next, we simulated IL-10 overexpression and knock-out conditions. Overexpression of IL-10 led to decrease in NFĸB and in all the cytokines examined while knock-out of IL-10 in the model relieved the inhibition on NFĸB and resulted in increase in NFĸB and in pro-inflammatory cytokines downstream (Figure 6 and Figure S5 in Methods S1).

### Temporal Analysis of GEO Data

**Figure 7:**
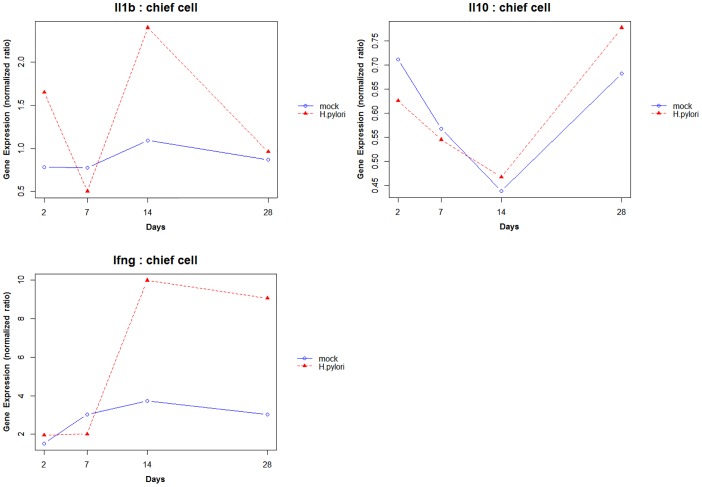
Trajectories of cytokines in mock-infected and *H. pylori* infected mice from GEO microarray dataset. (A) IL-1β, (B) IL-10 and (C) IFNγ for day 2, 7, 14 and 28 from chief cell of mock-infected and *H. pylori* infected mice. The temporal profiles indicate that these cytokines potentially display a cyclic expression pattern in response to *H. pylori* infection.

We also analyzed a microarray time-series dataset, available through GEO database. This data allowed us to study the temporal profile of IL-1β, IL-10 and IFNγ for both mock-infected and *H. pylori* infected mice. Figure 7 shows the trajectory of the three cytokines in chief cell for day 2, 7, 14 and 28. This time based analysis of the data suggests that these cytokines show a cyclic behavior as opposed to a linear trend. Figure S6 in Methods S1 shows the profiles for these cytokines in parietal and pit cells.

### Sensitivity and Stability Analysis

Our weakly non-linear system, modelled using a set of ordinary differential equations (ODE), can be viewed as a linear time-invariant system in the neighborhood of equilibrium and can be further studied using standard methods of analysis. In particular, the eigen-structure of the system can be used to algebraically identify the system behavior. For example, oscillations, a common result of negative feedback loops in biology, can be deduced from the complex eigenvalues. The damped oscillatory nature of our model, for example, depends on the negative inhibition of NFĸB by IL-10 and it is deduced from the complex eigenvalues (with negative real part) of the system's response. Removal of this inhibitory link in the model results in steady state behavior (Figure S7 in Methods S1) – which can be mathematically identified with the vanishing imaginary part of the eigenvalues. A sensitivity analysis is also performed to find the range of parameters across which the model displays damped oscillations. These results indicate that the cyclic behavior of the model remains viable within a large, biologically feasible, parameter region and is not limited to specific parameters and concentration values. Jacobian matrix and eigenvalues for the model were calculated using simulation tool – Copasi [83]. The parameters and concentrations that have the maximum influence on the imaginary part of eigenvalues were selected and varied over a wide range of values. The results presented in Table S5 in Methods S1 show that the model exhibits oscillatory trend even when original value of key parameters are increased or decreased by 50%. Further details of sensitivity analysis are provided in MethodsS1.

## Discussion

*H. pylori* is associated with high risk of gastric diseases including chronic gastritis, gastric ulcer and stomach cancer [84]. Experimental evidence has shown that *H. pylori* activates pro-inflammatory Th1-dependent cytokines such as IL-12, IFNγ, IL-1β, TNFα [85]–[88]. Often, immune system succeeds in decreasing *H. pylori* numbers but the bacteria is not completely eradicated [86], [89] and a constant low level of infection and chronic inflammation may ensue. In recent years, SHH has emerged as an important player in acute immune response to *H. pylori* [15]. But the relationship between SHH and cytokines remains unexplored. Our analysis shows that SHH positively regulates expression of cytokines like IL-12, IL-1β, MIP-2, IL-10, however the mechanism remains unclear.

We developed a simple mathematical model of the cytokines and SHH activated during *H. pylori* infection. Our model captures the positive regulation of cytokines by SHH through an intermediate model species ‘X’. We speculate the following mechanism through which SHH may play a key role in regulating the cytokines during *H. pylori* infection. We have recently shown that SHH acts as a macrophage chemoattractant in response to *H. pylori* infection [15]. It has been reported by independent studies that monocytes and macrophages treated with *H. pylori* secrete IL-1β, IL-12, IL-10, TNFγ, IL-6 [47]–[49]. Thus the positive influence on the expression of cytokines by SHH, predicted by our qPCR data may be mediated through recruitment of macrophage by SHH to the gastric epithelium. IL-12 secreted in turn activates release of IFNγ from natural killer cells [52], [53]. In future, it will be interesting to experimentally test if treatment of *H. pylori* stimulated macrophages, with SHH can further enhance the expression of above mentioned cytokines. Results from such experiments can help to identify unknown molecule ‘X’ in the current model and reduce the existing knowledge gap between SHH and cytokines activated during *H. pylori* infection.

A key advantage of *in-silico* modeling is that it facilitates the investigation of perturbations on a scale and extent that would be challenging and expensive to achieve on the bench top in an expedient manner. To understand the role of the anti-inflammatory cytokine IL-10 in our model circuit, we simulated overexpression and knock-out IL-10 conditions in the model. Our *in-silico* outcomes qualitatively matched published results of similar experiments. Robinson *et al* studied the effect of IL-10 in gastric epithelial cells (AGS cell line) [60] where they showed that addition of recombinant human IL-10 to co-cultures of *H. pylori* strain 60190 with AGS cells caused decrease in nuclear NFĸB and IL-8. Similar decreasing trends in NFĸB and MIP-2 (murine homologue of human IL-8) were observed with increase of IL-10 in our model (Figure 6 A, B). Knock-out of IL-10 in our model relieved the inhibition on NFĸB and resulted in increase in NFĸB and in pro-inflammatory cytokines downstream. Figure 6 C shows increase in pro-inflammatory cytokine IFNγ with IL-10 KO as compared to that in WT, thus suggesting the potential protective role of IL-10 against *H. pylori* mediated gastritis. Bodger *et al* have also suggested that IL-10 secretion during *H. pylori* infection may serve a protective role, reducing local tissue damage caused by inflammation [90]. Ismail et al have shown that *Helicobacter felis* infection in IL-10 knock-out in mice resulted in a higher inflammation score and severe gastritis as compared to that in infected wild type mice [11]. They also cultured splenocytes from control uninfected and *H. felis*-infected WT and IL-10 KO mice, with sonicated *H. felis* Ag and the culture supernatants were evaluated for the concentration of IFN-γ. Splenocytes from *H. felis*-infected WT mice produced low levels of IFN-γ while splenocytes from *H. felis*-infected IL-10^−/−^ mice produced large amounts of IFN-γ [11]. Future experiments evaluating the effect of IL-10 on pro-inflammatory cytokines (like IL-12, IL-1β, IFNγ, IL6, TNFα) and SHH can enhance our current understanding about the role of IL-10 in gastric mucosa during *H. pylori* infection.

The model also helps to bring out emergent properties of the network, which can guide future experimental studies and enhance our current understanding of the system. Our model suggests that NFĸB, SHH and the cytokines engage in a feedback loop which can result in damped oscillations. Temporal analysis of cytokines from a microarray Geo dataset also indicates that IL-1β, IFNγ and IL-10 from *H. pylori* infected mice, show a cyclic behavior rather than a linear trend. However, time course experiments capturing dynamic trajectories of expression of genes involved in the network are required to validate this hypothesis. Based on our preliminary results from analysis of Geo dataset, we propose that experiments capturing expression of cytokines and other genes at different time points (on scale of days) can inform about time dependent variation in expression of genes and also help to study the phase of correlations among pairs of genes and understand their relationships. We use the mathematical model as a tool to gain insights into cytokine and SHH relationship during *H. pylori* infection and as a hypothesis generating tool to predict host responses that may be associated with gastric disease or clinical treatments that may provide a better outcome.

## Supporting Information

File S1 **Supporting table.** Table S6, Raw qPCR data.
(CSV)

Methods S1 **Supporting files.** Figure S1, Interaction Map of signaling pathways activated in host stomach in response to *H. pylori*. *H. pylori* virulence factors (CagA, VacA and PGN, shown in orange) activate cascade of signaling pathways in host gastric epithelium that leads to nuclear translocation of NFĸB. NFĸB further activates IL8/MIP-2 and SHH. Immune response to the bacteria involves recruitment of monocytes to gastric epithelium where they secrete cytokines like IL-12, IL-1β, TNFα, IL6, IL10 and IL8. Blue arrows show activation while red lines represent inhibition. The network was built using Cytoscape using information based on current literature. However, the current knowledge does not inform about any role of SHH in regulation of cytokines as suggested by our analysis. Figure S2, Effect of *H. pylori* infection on IFNγ expression on day 180 in wild-type (WT) and parietal cell-specific SHH KO (PC-SHH-KO) mice. RNA was extracted from stomachs of uninfected (-HP) and *H. pylori*-infected (+HP) wild type and parietal cell specific SHH knock-out mice 180 days post-inoculation and expression of IFNγ was measured by qPCR. (A) Interaction plot between infection status and genotype. P-value for interaction between infection and genotype was calculated by two-way ANOVA test. Y-axis: Negative dCT value of IFNγ, X-axis: infection status, trace-factor: genotype. (B) Fold change in expression of IFNγ relative to uninfected condition in WT and PC-SHH^KO^ mice. Two-way ANOVA test was performed, followed by Bonferroni test to compare uninfected (-HP) with infected group (+HP) in each genotype. Bars represent the mean ± SEM, n = 4 per group. Figure S3, *In-silico* SHH KO results in model lacking the predicted link show no change in cytokines as comared to WT. SHH KO condition was simulated by setting SHHi to zero. Graph A-F shows profiles of (A) SHH (B) IL-1β (C) IL-12 (D) IFNγ (E) MIP2 (F) IL10. Wild type condition (SHHi = 1) is shown in yellow and *in-silico* SHH KO condition (SHHi  = 0) is represented in red. Cytokines show no change (orange color is observed as a result of overlap of red and yellow lines). Figure S4, *In-silico* temporal profiles of (A) IL-12 and (B) IFNγ in absence and presence of *H. pylori*. Figure S5, *In-silico* IL-10 knock-out and overexpression. Effect of IL-10 knock-out and overexpression on (A) IL-1β (B) IL-12 and (C) SHH. Wild type condition (IL10i  = 1) is shown in black, *in-silico* IL-10 knok-out (IL10i  = 0) in red and *in-silico* IL-10 overexpression (IL10i  = 10) in green. Figure S6, Trajectory of IL-1β, IL-10 and IFNγ for day 2, 7, 14 and 28 for parietal and pit cell from mock-infected and H.pylori infected mice. The temporal profiles indicate that these cytokines show non-uniform behavior than a linear trend. Figure S7, Temporal profiles of model species in absence of negative feedback on NFĸB by IL-10. The damped oscillatory nature of the model depends on the negative inhibition of NFĸB by IL-10. Removal of this inhibitory link in the model results in steady state behavior. Table S1, Model Assumptions. Table S2, Mathematical equations used in the model. Table S3, Species' parameters used in the model. Table S4, Kinetic parameters used in the model. Table S5, Sensitivity Analysis of model parameters for damped oscillations. Key parameters and their range for which the model shows damped oscillations.
(DOC)
