# Supplementary material for: Crosstalks between Cytokines and Sonic Hedgehog in Helicobacter pylori Infection: A Mathematical Model
Source: PLoS One. 2014 Nov 3;9(11):e111338. doi: 10.1371/journal.pone.0111338 (PMC4218723; doi:10.1371/journal.pone.0111338)
Supplement: Methods S1 — Supporting files. Figure S1, Interaction Map of signaling pathways activated in host stomach in response to H. pylori. H. pylori virulence factors (CagA, VacA and PGN, shown in orange) activate cascade of signaling pathways in host gastric epithelium that leads to nuclear translocation of NFĸB. NFĸB further activates IL8/MIP-2 and SHH. Immune response to the bacteria involves recruitment of monocytes to gastric epithelium where they secrete cytokines like IL-12, IL-1β, TNFα, IL6, IL10 and IL8. Blue arrows show activation while red lines represent inhibition. The network was built using Cytoscape using information based on current literature. However, the current knowledge does not inform about any role of SHH in regulation of cytokines as suggested by our analysis. Figure S2, Effect of H. pylori infection on IFNγ expression on day 180 in wild-type (WT) and parietal cell-specific SHH KO (PC-SHH-KO) mice. RNA was extracted from stomachs of uninfected (-HP) and H. pylori-infected (+HP) wild type and parietal cell specific SHH knock-out mice 180 days post-inoculation and expression of IFNγ was measured by qPCR. (A) Interaction plot between infection status and genotype. P-value for interaction between infection and genotype was calculated by two-way ANOVA test. Y-axis: Negative dCT value of IFNγ, X-axis: infection status, trace-factor: genotype. (B) Fold change in expression of IFNγ relative to uninfected condition in WT and PC-SHHKO mice. Two-way ANOVA test was performed, followed by Bonferroni test to compare uninfected (-HP) with infected group (+HP) in each genotype. Bars represent the mean ± SEM, n = 4 per group. Figure S3, In-silico SHH KO results in model lacking the predicted link show no change in cytokines as comared to WT. SHH KO condition was simulated by setting SHHi to zero. Graph A-F shows profiles of (A) SHH (B) IL-1β (C) IL-12 (D) IFNγ (E) MIP2 (F) IL10. Wild type condition (SHHi = 1) is shown in yellow and in-silico SHH KO condition (SHHi = 0) is repre [file pone.0111338.s002.doc]

**Supporting Information**

**Title:** **C**rosstalks between cytokines and sonic hedgehog in *Helicobacter pylori* infection: A mathematical model.

**Materials & Methods**

# Animal Model

# The mouse model with parietal cell-specific deletion of SHH (PC-SHH^KO^) was generated as previously described [1]. Genotyping was based on polymerase chain reaction primers and protocols described in Clausen *et al.* [2] and Long *et al.* [3]. Mice were genotyped for LyzsCre using primer pair (5’- CCC AGA AAT GCC AGA TTA CG -3’) and (5’- CTT GGG CTG CCA GAA TTT CTC -3’) and for wild-type Lyzs gene using primer pair (5’- TTA CAG TCG GCC AGG CTG AC -3’) and (5’- CTT GGG CTG CCA GAA TTT CTC -3’). PCR thermocycler conditions for LyzsCre and Lyzs gene are as follows: 94° C (1 cycle, 3 minutes), 35 cycles of 94° C (30 seconds), 62° C (1 minute), and 72° C (1 minute), then 1 final cycle of 72° C for 2 minutes. All mice were 8 weeks of age when inoculated. All mouse studies were approved by the University of Cincinnati Institutional Animal Care and Use Committee (IACUC) that maintains an American Association of Assessment and Accreditation of Laboratory Animal Care (AAALAC) facility.

*Helicobacter pylori* culture conditions and quantification

*Helicobacter pylori* SS1 (Sydney strain 1) (kindly donated by Dr. KA Eaton, University of Michigan) bacteria were grown in brucella broth supplemented with 5% fetal calf serum in a humidified microaerophilic chamber (BBL Gas System, with CampyPak Plus packs, BD Microbiology, Sparks, MD) in a shaking incubator at 37^o^C for 16 hours. Bacteria were harvested and used to inoculate mouse stomachs by oral intubation over 3 consecutive days with 10^8^ *H. pylori* bacteria per 200 μl of brucella broth. Control mice (uninfected group) received 200 μl of brucella broth over 3 consecutive days. *H. pylori* colonization was quantified using the culture method previously published [29]. Briefly, the wet weight of gastric tissue collected from uninfected and infected control and PC-SHH^KO^ groups was measured. Tissue was then homogenized in 1 ml saline and dilutions ranging from 1/10 to 1/1000 were spread on blood agar plates containing *Campylobacter* Base Agar (Fischer Scientific), 5% horse blood (BD Diagnostic Systems), 5μg/ml vancomycin and 10μg/ml trimethoprim. Plates were incubated for 5-7 days at 37^o^C in a humidified microaerophilic chamber. Single colonies from these plates tested positive for urease (BD Diagnostic Systems), catalase (using 3% H_2_O_2_) and oxidase (DrySlide, BD Diagnostic Systems). Colonies were counted and data normalized using the tissue wet weight and expressed and colony forming units (CFU)/g tissue.

Quantitative real-time RT-PCR (qPCR)

Total RNA was isolated from stomachs of uninfected and infected control and PC-SHH^KO^ mice. The High Capacity cDNA Reverse Transcription Kit was used for cDNA synthesis from 100ng of RNA following the recommended protocol (Applied Biosystems). Pre-designed real-time PCR assays were purchased for the following genes (Applied Biosystems): Shh (Mm00436528_m1), IFNγ (Mm01168134_m1), IL-1β (Mm01336189_m1) and mouse GAPDH (20X) (4352932-0803020). PCR amplifications were performed in a total volume of 20 μl, containing 20X TaqMan Expression Assay primers, 2X TaqMan Universal Master Mix (Applied Biosystems, TaqMan® Gene Expression Systems) and cDNA template. Each PCR amplification was performed in duplicate wells in a StepOne™ Real-Time PCR System (Applied Biosystems), using the following conditions: 50^o^C 2 minutes, 95^o^C 10 minutes, 95^o^C 15 seconds (denature) and 60^o^C 1 minute (anneal/extend) for 40 cycles. IL-12 expression was quantified using specific primers as previously published for IL-12: Forward- 5’- GGA AGC ACG GCA GCA GAA TA-3’ and Reverse- 5’- AAC TTG AGG GAG AAG TAG GAA TGG -3’ [4] using the SYBR Green PCR Master Mix and protocol (Applied Biosystems). Raw qPCR data is provided as Table S6 in File S1.

Sensitivity Analysis

Steady state or oscillatory behavior of a system can be determined by eigenvalues derived from Jacobian matrix of that system. Eigenvalues (λ) are complex numbers with real and imaginary part and inform about stability of the system. Imaginary part of eigenvalue determines if the system will oscillate while real part determines whether the amplitude of oscillations increase or decrease with time. When λ has negative real part and zero imaginary part, the system decays exponentially; for λ with at least one positive real part and nonzero imaginary part, the system will increase exponentially. If λ has negative real part and nonzero imaginary part, the system exhibits damped oscillations; if λ has zero real part and nonzero imaginary part, the system shows sustained oscillations [5,6].

We used the above mathematical principles to understand the behavior of our model. To ensure that the cyclic nature of our mathematical model is not depended on specific parameter and concentration values, but rather observed for a wide range of biologically feasible values, we performed sensitivity analysis. First, we determined the parameter and concentration values which had maximum influence on the imaginary part of eigenvalues. These parameter values were varied (+- 50% or more) to assess the boundary value for the parameter beyond which the imaginary part of eigenvalues will be zero and the system moves towards stable point. The results presented in Table 4 show that the model exhibits oscillatory trend even when key parameters are varied over a range of 10 folds.

**References**

1. Xiao C, Ogle S a, Schumacher M a, Orr-Asman M a, Miller ML, et al. (2010) Loss of parietal cell expression of Sonic hedgehog induces hypergastrinemia and hyperproliferation of surface mucous cells. Gastroenterology 138: 550–61, 561.e1–8. doi:10.1053/j.gastro.2009.11.002.

2. Clausen BE, Burkhardt C, Reith W, Renkawitz R, Förster I (1999) Conditional gene targeting in macrophages and granulocytes using LysMcre mice. Transgenic Res 8: 265–277.

3. Long F, Zhang XM, Karp S, Yang Y, McMahon AP (2001) Genetic manipulation of hedgehog signaling in the endochondral skeleton reveals a direct role in the regulation of chondrocyte proliferation. Development 128: 5099–5108.

4. Lehmann J, Bellmann S, Werner C, Schröder R, Schütze N, et al. (2001) IL-12p40-dependent agonistic effects on the development of protective innate and adaptive immunity against Salmonella enteritidis. J Immunol 167: 5304–5315.

5. Ferrell JE, Tsai TY-C, Yang Q (2011) Modeling the cell cycle: why do certain circuits oscillate? Cell 144: 874–885. doi:10.1016/j.cell.2011.03.006.

6. A.K. Konopka (2006) Systems Biology: Principles, Methods, and Concepts.

7. Novak B, Pataki Z, Ciliberto A, Tyson JJ (2001) Mathematical model of the cell division cycle of fission yeast. Chaos 11: 277–286. doi:10.1063/1.1345725.

8. Ciliberto A, Petrus MJ, Tyson JJ, Sible JC (2003) A kinetic model of the cyclin EyCdk2 developmental timer in Xenopus laevis embryos. Biophys Chem 104: 573–589. doi:10.1016/S0301-4622.

9. Chen KC, Calzone L, Csikasz-nagy A, Cross FR, Novak B, et al. (2004) Integrative Analysis of Cell Cycle Control in Budding Yeast □. Mol Biol Cell 15: 3841–3862. doi:10.1091/mbc.E03.

**Supporting Information Figures**


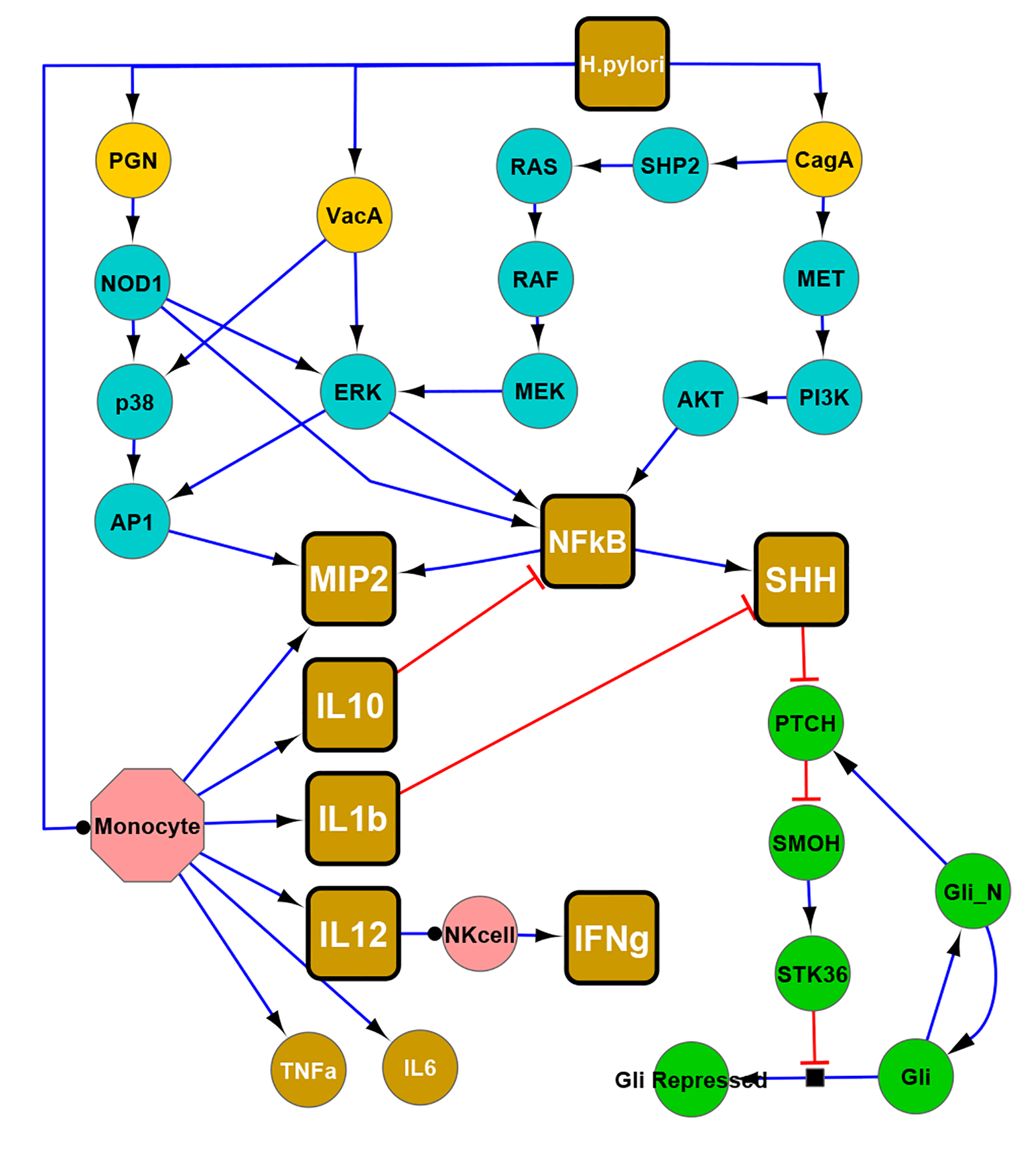


**Figure S1**. **Interaction Map of signaling pathways activated in host stomach in response to *H. pylori*.** *H. pylori* virulence factors (CagA, VacA and PGN, shown in orange) activate cascade of signaling pathways in host gastric epithelium that leads to nuclear translocation of NFĸB. NFĸB further activates IL8/MIP-2 and SHH. Immune response to the bacteria involves recruitment of monocytes to gastric epithelium where they secrete cytokines like IL-12, IL-1β, TNFα, IL6, IL10 and IL8. Blue arrows show activation while red lines represent inhibition. The network was built using Cytoscape using information based on current literature. However, the current knowledge does not inform about any role of SHH in regulation of cytokines as suggested by our analysis.

**
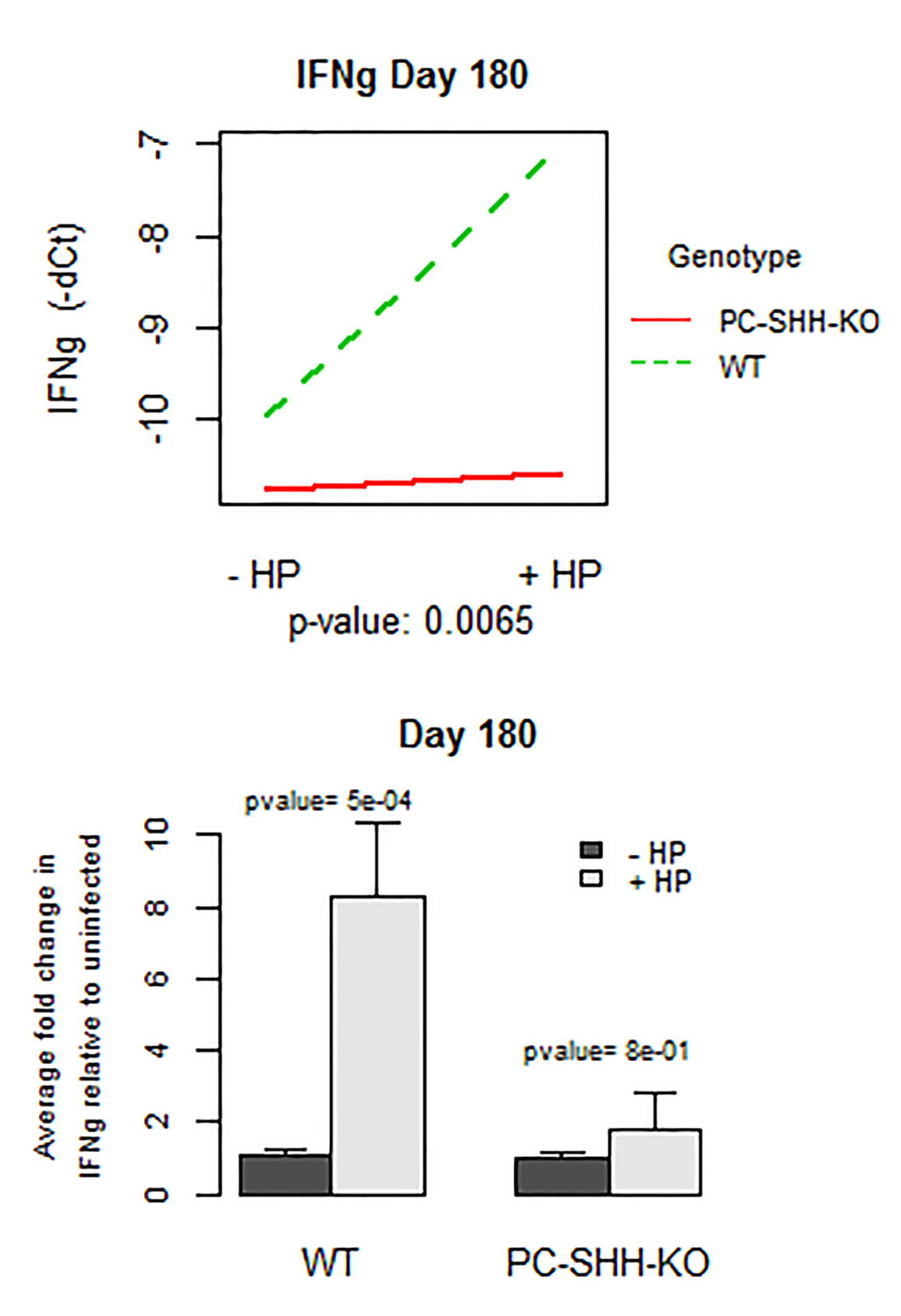
**

**Figure S2. Effect of *H. pylori* infection on IFNγ expression on day 180 in wild-type (WT)** **and parietal cell-specific SHH KO (PC-SHH-KO) mice.** RNA was extracted from stomachs of uninfected (-HP) and *H. pylori*-infected (+HP) wild type and parietal cell specific SHH knock-out mice 180 days post-inoculation and expression of IFNγ was measured by qPCR. (A) Interaction plot between infection status and genotype. P-value for interaction between infection and genotype was calculated by two-way ANOVA test. Y-axis: Negative dCT value of IFNγ, X-axis: infection status, trace-factor: genotype. (B) Fold change in expression of IFNγ relative to uninfected condition in WT and PC-SHH^KO^ mice. Two-way ANOVA test was performed, followed by Bonferroni test to compare uninfected (-HP) with infected group (+HP) in each genotype. Bars represent the mean ±SEM, n=4 per group.

**
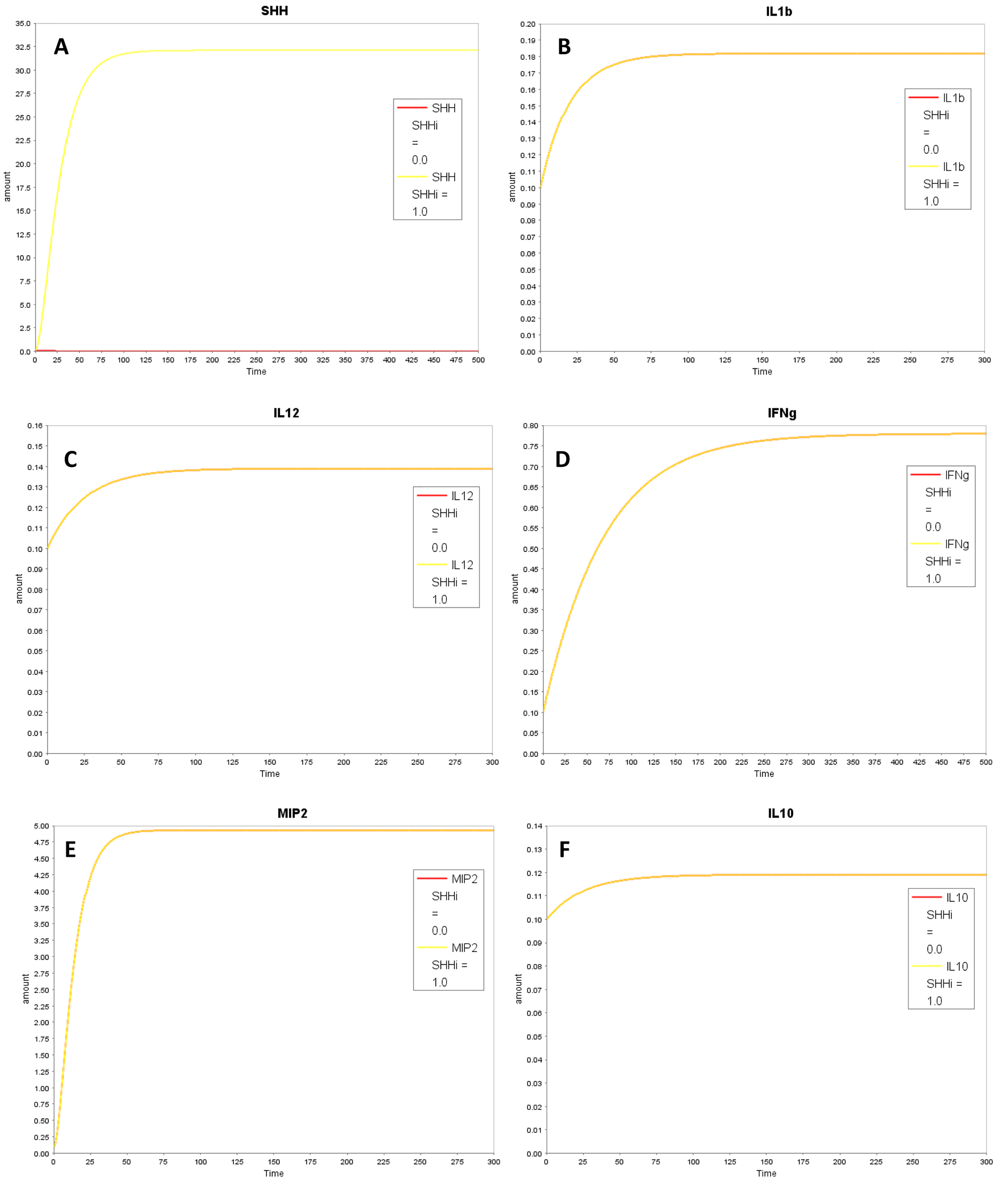
**

**Figure S3. *In-silico* SHH KO results in model lacking the predicted link show no change in cytokines as comared to WT.** SHH KO condition was simulated by setting SHHi to zero. Graph A-F shows profiles of (A) SHH (B) IL-1β (C) IL-12 (D) IFNγ (E) MIP2 (F) IL10. Wild type condition (SHHi=1) is shown in yellow and *in-silico* SHH KO condition (SHHi=0) is represented in red. Cytokines show no change (orange color is observed as a result of overlap of red and yellow lines).

**
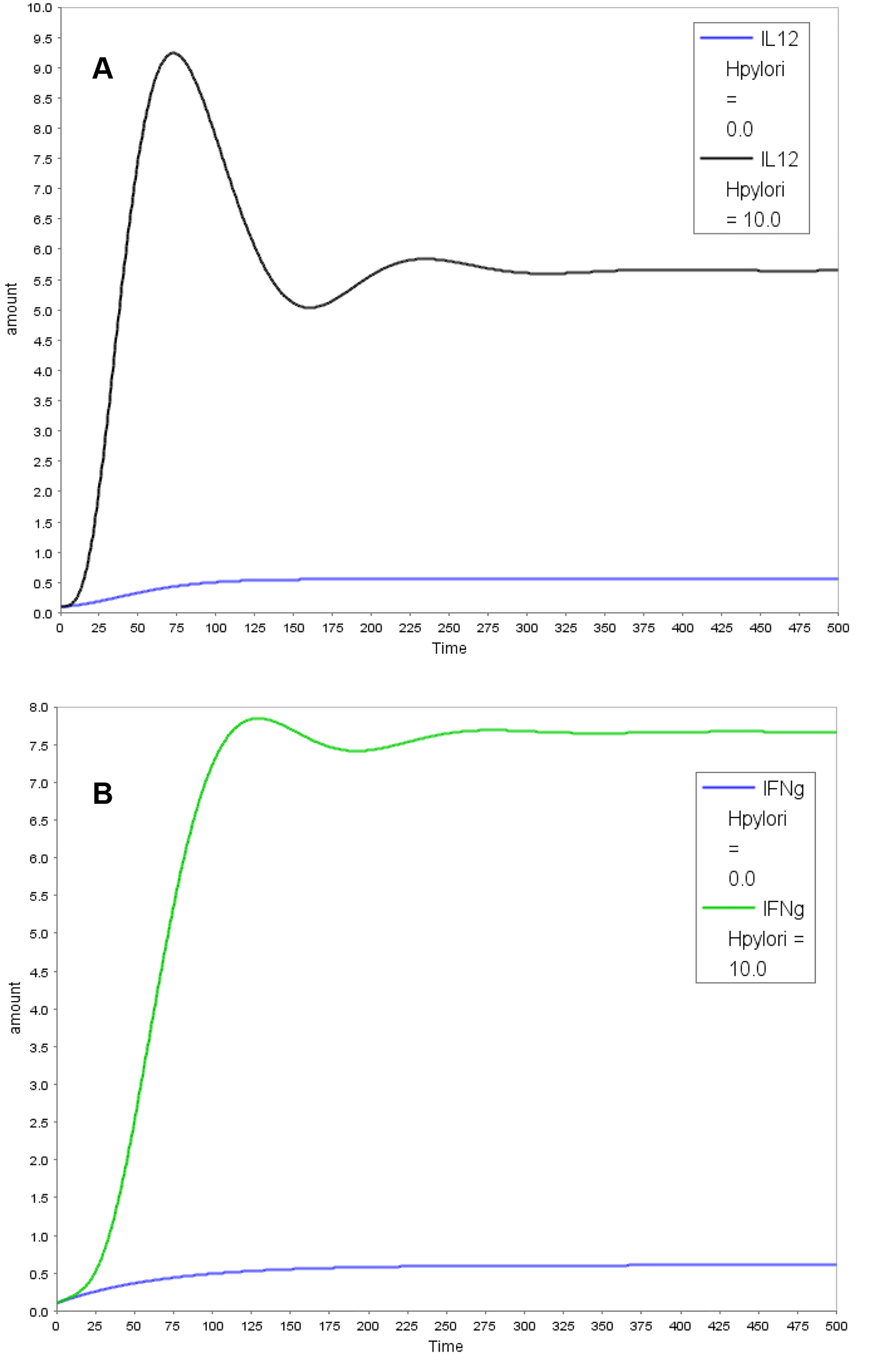
**

**Figure S4. *In-silico*** t**emporal profiles of (A) IL-12 and (B) IFNγ in absence and presence of *H. pylori*.**

**
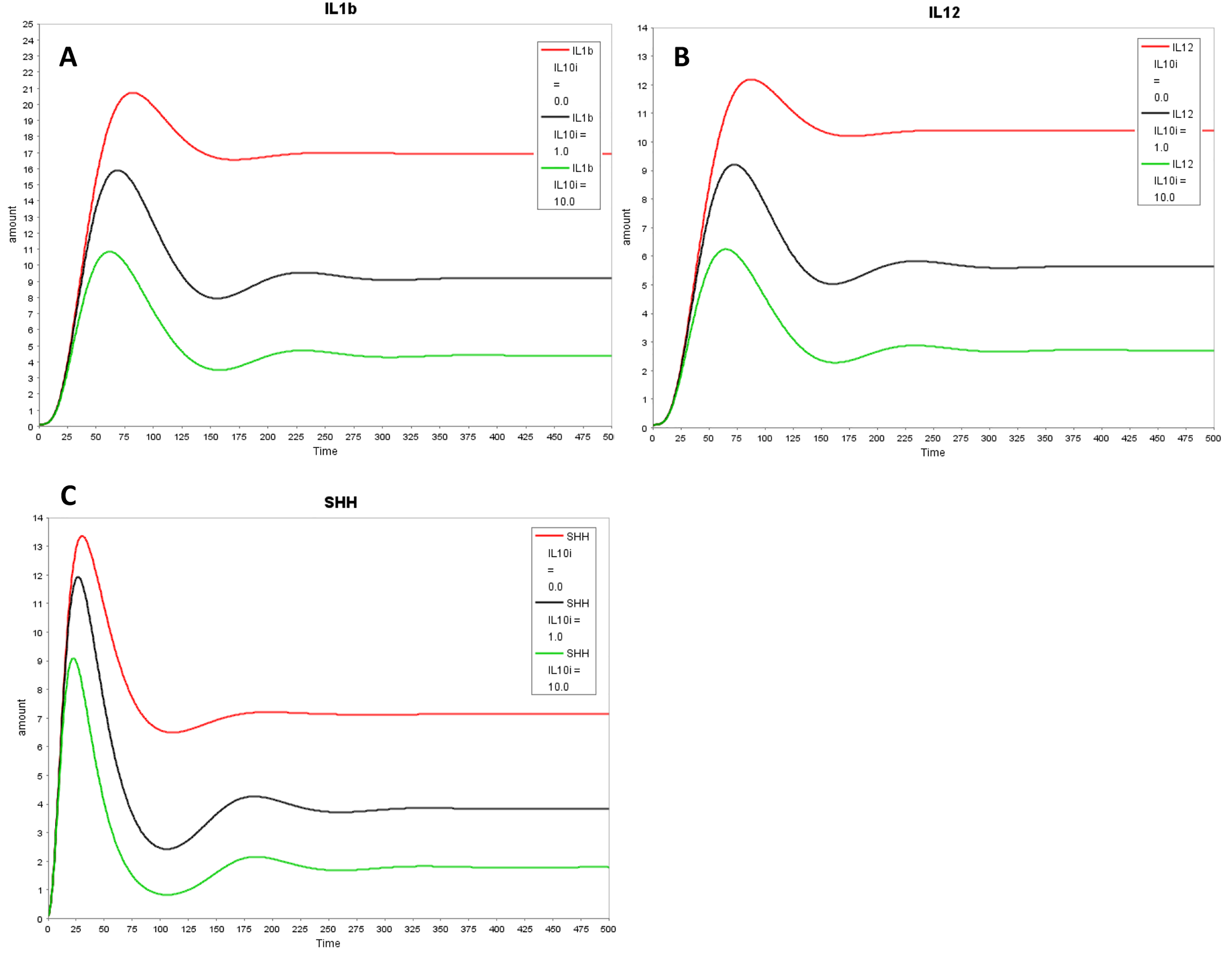
**

**Figure S5. *In-silico* IL-10 knock-out and overexpression**. Effect of IL-10 knock-out and overexpression on (A) IL-1β (B) IL-12 and (C) SHH. Wild type condition (IL10i=1) is shown in black, *in-silico* IL-10 knok-out (IL10i=0) in red and *in-silico* IL-10 overexpression (IL10i=10) in green.


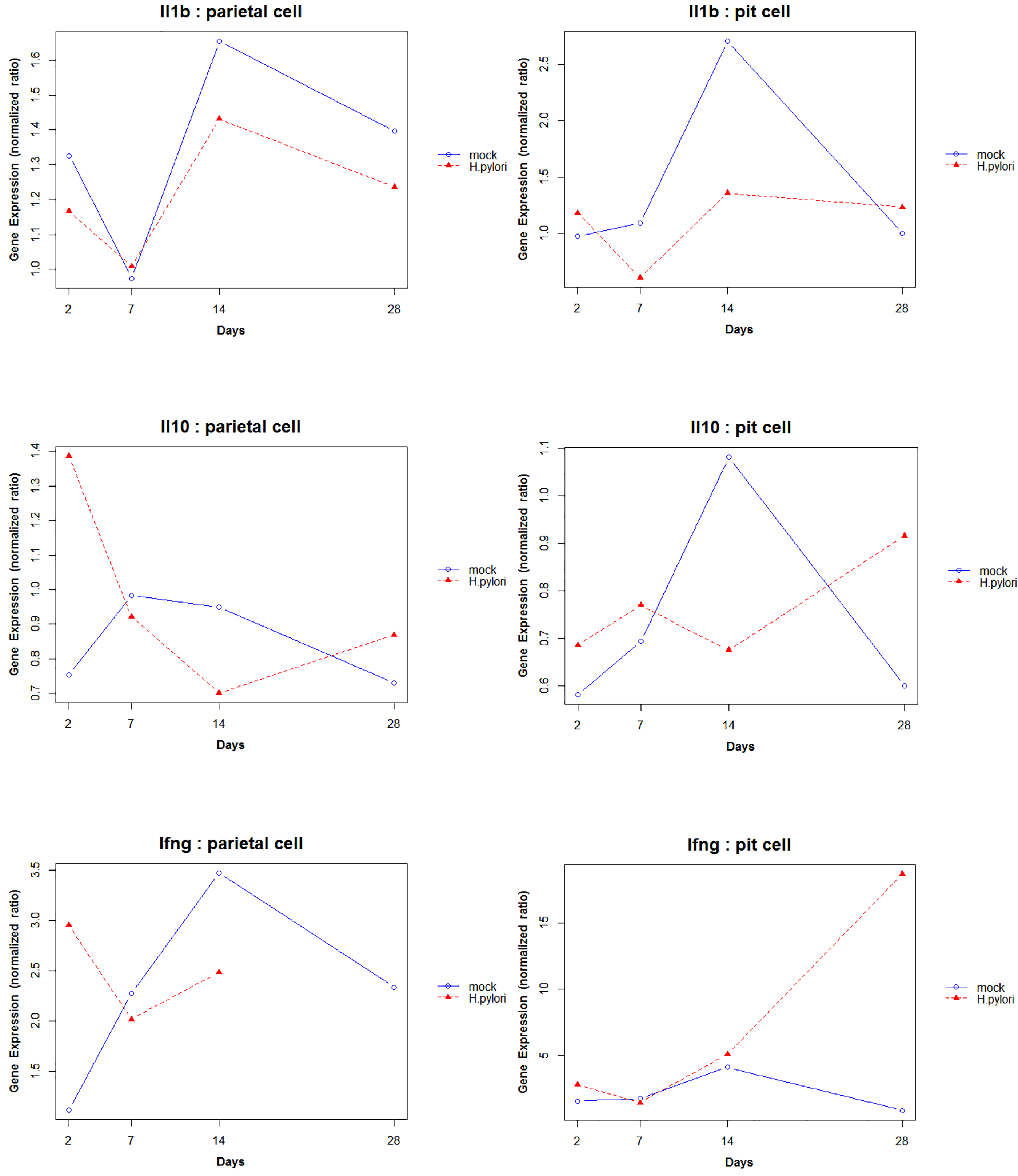


**Figure S6**. Trajectory of IL-1β, IL-10 and IFNγ for day 2, 7, 14 and 28 for parietal and pit cell from mock-infected and H.pylori infected mice. The temporal profiles indicate that these cytokines show non-uniform behavior than a linear trend.

**
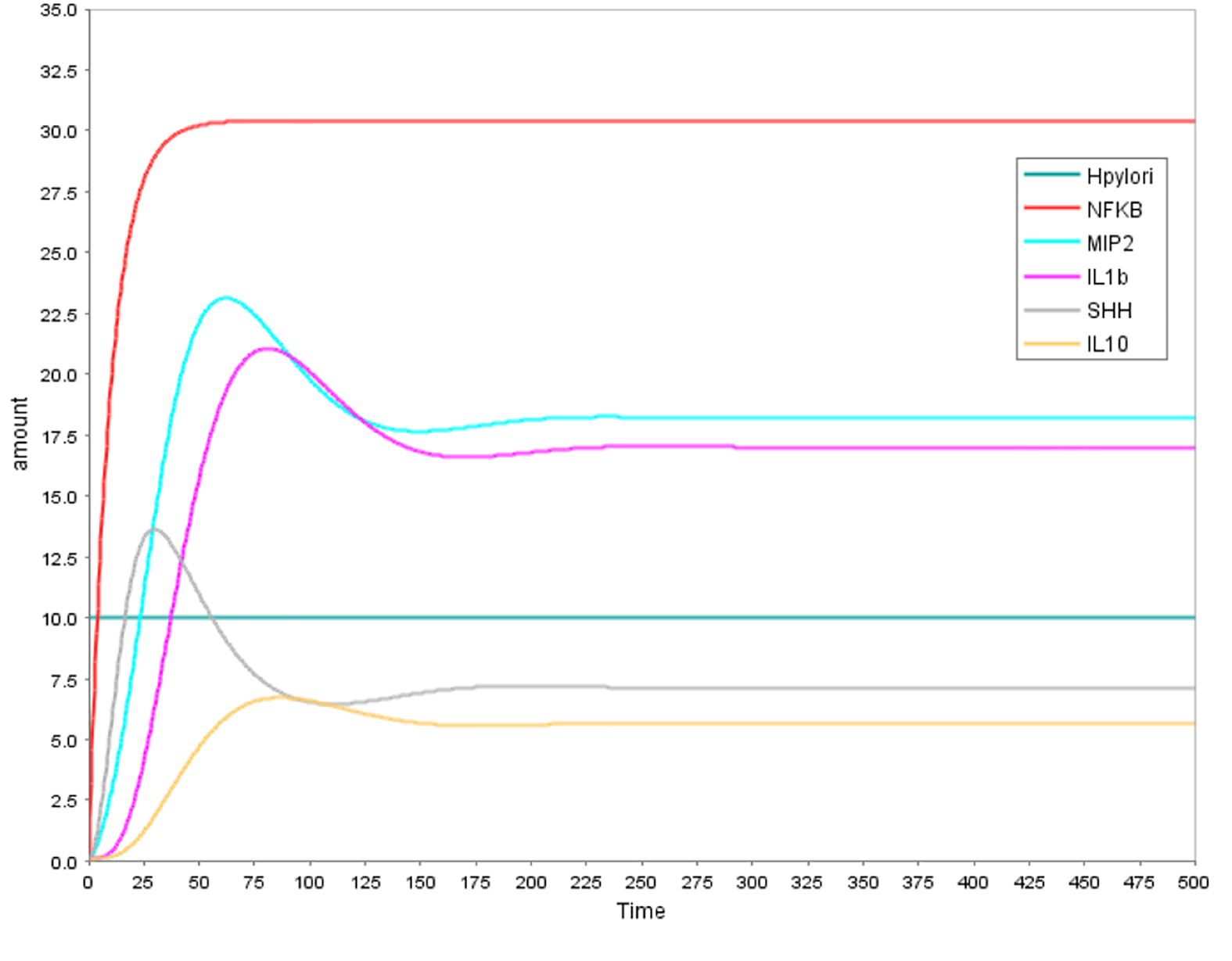
**

**Figure S7**. **Temporal profiles of model species in absence of negative feedback on NFĸB by IL-10.** The damped oscillatory nature of the model depends on the negative inhibition of NFĸB by IL-10. Removal of this inhibitory link in the model results in steady state behavior.

**Tables**

**Table S1.** **Model Assumptions**

1. Model compresses the details of a complex pathway into a “black box”, which can be scaled to larger, detailed components when needed. For example activation (nuclear transport) of NFkB involves activation of ERK, AKT pathways by *H. pylori* virulence factors which includes many steps, has been compressed to a single step. Similarly, cellular details of transcription, translation and post translation modifications are not shown. The focus of this model is to provide a high level understanding of cytokine-SHH circuit during *H. pylori* infection.
2. Equations were based on Michaelis Menten and mass action kinetics.
3. “Time” and “concentration” are expressed in arbitrary units as we do not have enough experimental data to confidently measure proteins’ concentration or frequency of oscillations.
4. Kinetic parameters initially selected for the model, represented a range of biologically feasible values [7–9]. Subsequent computational optimization (iterative trial and error) was used to select the parameter set that best satisfies the trends observed in experimental data and are within biologically relevant limits.
5. The interaction map focuses on key signaling pathways activated in gastric epithelium and in macrophages, by *H. pylori* virulence factors. Pathways in other immune cells are currently not included but in future it will be of great value to include them in both interaction map and model.
6. A constant, steady state level of *H. pylori* is shown in current model.

**Table S2. Mathematical equations used in the model.**

| **Reaction Id** | **Reactants** | **Products** | **Modifiers** | **Equation** |
| --- | --- | --- | --- | --- |
| re1 | NFKBi | NFKB | Hpylori,IL10 | kcat1 * Hpylori * NFKBi / (km1 * (1 + IL10 / Ki1) + NFKBi) |
| re2 | NFKBi | NFKB |  | v1 * NFKBi / (k1 + NFKBi) |
| re3 | NFKB | NFKBi |  | v1 * NFKB / (k1 + NFKB) |
| re4 | MIP2i | MIP2 |  | v1 * MIP2i / (k1 + MIP2i) |
| re5 | MIP2i | MIP2 | NFKB | kcat5 * NFKB * MIP2i / (km5 + MIP2i) |
| re6 | MIP2i | MIP2 | X | kcat6 * X * MIP2i / (km6 + MIP2i) |
| re7 | SHHi | SHH | NFKB,IL1b | kcat7 * NFKB * SHHi / (km7 * (1 + IL1b / Ki7) + SHHi) |
| re8 | SHHi | SHH |  | v1 * SHHi / (k1 + SHHi) |
| re9 | Xi | X | SHH | kcat9 * SHH * Xi / (km9 + Xi) |
| re10 | IL1bi | IL1b | X | kcat10 * X * IL1bi / (km10 + IL1bi) |
| re11 | IL1bi | IL1b |  | v1 * IL1bi / (k1 + IL1bi) |
| re12 | IL12i | IL12 | X | kcat12 * X * IL12i / (km8 + IL12i) |
| re13 | IL12i | IL12 |  | v1 * IL12i / (k1 + IL12i) |
| re14 | IL10i | IL10 | X | kcat14 * X * IL10i / (km14 + IL10i) |
| re15 | IL10i | IL10 |  | v1 * IL10i / (k1 + IL10i) |
| re16 | IFNgi | IFNg | IL12,Hpylori | kcat16 * Hpylori * IL12 * IFNgi / (km16 + IFNgi) |
| re17 | IFNgi | IFNg |  | v1 * IFNgi / (k1 + IFNgi) |
| re18 | NFKB | Sink |  | NFKB * k1 |
| re19 | MIP2 | Sink |  | MIP2 * k1 |
| re20 | SHH | Sink |  | SHH * k1 |
| re21 | IL1b | Sink |  | IL1b * k1 |
| re22 | IL12 | Sink |  | IL12 * k1 |
| re23 | IL10 | Sink |  | IL10 * k1 |
| re24 | IFNg | Sink |  | IFNg * k1 |
| re25 | X | Sink |  | X * k1 |

**Table S3. Species’ parameters used in the model.**

| **Species Name** | **Initial Quantity** | **Constant** |
| --- | --- | --- |
| Hpylori | 10 | TRUE |
| NFKB | 0.1 | FALSE |
| NFKBi | 1 | TRUE |
| MIP2 | 0.1 | FALSE |
| MIP2i | 1 | TRUE |
| IL1b | 0.1 | FALSE |
| SHHi | 1 | TRUE |
| SHH | 0.1 | FALSE |
| IL10 | 0.1 | FALSE |
| X | 0 | FALSE |
| IL1bi | 1 | TRUE |
| Xi | 1 | TRUE |
| IL10i | 1 | TRUE |
| IL12i | 1 | TRUE |
| IL12 | 0.1 | FALSE |
| IFNgi | 1 | TRUE |
| IFNg | 0.1 | FALSE |
| Sink | 0 | TRUE |

**Table S4. Kinetic parameters used in the model.**

| **Reaction Id** | **Parameter Name** | **Value** |
| --- | --- | --- |
| (re1) | km1 | 0.521 |
| (re1) | kcat1 | 0.516 |
| (re1) | Ki1 | 0.702 |
| (re2) | v1 | 0.2 |
| (re2) | k1 | 0.5 |
| (re3) | v1 | 0.5 |
| (re3) | k1 | 1 |
| (re4) | v1 | 0.1 |
| (re4) | k1 | 2 |
| (re5) | kcat5 | 0.1 |
| (re5) | km5 | 2 |
| (re6) | kcat6 | 0.7 |
| (re6) | km6 | 4 |
| (re7) | kcat7 | 1.01 |
| (re7) | km7 | 16.25 |
| (re7) | Ki7 | 3.98 |
| (re8) | v1 | 0.01 |
| (re8) | k1 | 8 |
| (re9) | kcat9 | 0.211 |
| (re9) | km9 | 1.03 |
| (re10) | km10 | 10 |
| (re10) | kcat10 | 0.5 |
| (re11) | v1 | 0.1 |
| (re11) | k1 | 10 |
| (re12) | km8 | 8 |
| (re12) | kcat12 | 0.2 |
| (re13) | v1 | 0.05 |
| (re13) | k1 | 8 |
| (re14) | kcat14 | 0.253 |
| (re14) | km14 | 20.1 |
| (re15) | v1 | 0.1 |
| (re15) | k1 | 20 |
| (re16) | kcat16 | 0.03 |
| (re16) | km16 | 15 |
| (re17) | v1 | 0.1 |
| (re17) | k1 | 10 |
| (re18) | k1 | 0.1 |
| (re19) | k1 | 0.2 |
| (re20) | k1 | 0.05 |
| (re21) | k1 | 0.05 |
| (re22) | k1 | 0.04 |
| (re23) | k1 | 0.04 |
| (re24) | k1 | 0.015 |
| (re25) | k1 | 0.04 |

**Table S5.** **Sensitivity Analysis of model parameters for damped oscillations.** Key parameters and their range for which the model shows damped oscillations.

| **Parameter Name** | (re20) k1 | (re23) k1 | (re18) k1 | (re25) k1 | Hpylori |
| --- | --- | --- | --- | --- | --- |
| **Parameter Range** | 0.005 to 0.5 | 0.02 to 0.2 | 0.005 to 0.5 | 0.01 to 0.2 | 1 to 100 |
